# Switching from cigarette use to dual use with heated tobacco products and severe periodontitis risk

**DOI:** 10.3389/froh.2026.1765902

**Published:** 2026-01-26

**Authors:** Chihiro Shiota, Kenji Takeuchi, Yudai Tamada, Taro Kusama, Ken Osaka, Takahiro Tabuchi

**Affiliations:** 1 Department of International and Community Oral Health, Tohoku University Graduate School of Dentistry, Sendai, Japan; 2 Division of Statistics and Data Science, Liaison Center for Innovative Dentistry, Tohoku University Graduate School of Dentistry, Sendai, Japan; 3 Department of Preventive Medicine, Nagoya University Graduate School of Medicine, Aichi, Japan; 4 Division of Epidemiology, School of Public Health, Graduate School of Medicine, Tohoku University, Sendai, Japan

**Keywords:** cohort studies, periodontitis, risk factors, smoking, tobacco products

## Abstract

**Introduction:**

Severe periodontitis is a major global health concern, yet the impact of switching from combustible cigarettes to dual use of combustible cigarettes and heated tobacco products on periodontal status remains unclear. This study aimed to examine whether changes in smoking status influence the risk of severe periodontitis among Japanese adults.

**Methods:**

We conducted a longitudinal analysis using panel data from the Japan COVID-19 and Society Internet Survey. Participants were daily combustible cigarette smokers with periodontitis in 2021 and were followed through 2023. Smoking trajectories between 2021 and 2022 were categorized as: (1) continuous combustible cigarette smokers, (2) dual users (heated tobacco product-dominant), (3) dual users (combustible cigarette-dominant), and (4) quitters/reduced smokers. Severe periodontitis in 2023 was defined using validated self-reported criteria. Modified Poisson regression with robust variance was applied to estimate risk ratios and 95% confidence intervals (95% CIs), adjusting for potential confounders (age, sex, educational attainment, occupational status, marital status, equivalent income, alcohol consumption, diabetes mellitus status, periodontal pain, and smoking frequency).

**Results:**

A total of 193 participants (mean age 57.6 years; men 67.9%) were analyzed. Over 2 years, 61.1% developed severe periodontitis. Compared to continuous combustible cigarette smokers, the adjusted risk of severe periodontitis was 1.19 times (95% CI: 0.67–2.11), 1.41 times (95% CI: 1.04–1.91), and 0.99 times (95% CI: 0.76–1.28) higher for dual users (heated tobacco product-dominant), dual users (combustible cigarette-dominant), and quitters/reduced smokers, respectively. Sensitivity analyses using inverse probability weighting yielded consistent findings.

**Conclusion:**

Switching from daily combustible cigarette smoking to dual use with heated tobacco products may increase the risk of severe periodontitis. Combining combustible cigarettes with heated tobacco products, regardless of the frequency of combustible cigarette smoke, should not be considered a harm-reduction strategy in dental and public health practice.

## 1 Introduction

Severe periodontitis is one of the most prevalent dental issues and critical health problems globally. According to the Global Burden of Disease Study (2021), severe periodontitis ranked as the 31st-most prevalent condition among adults worldwide, with approximately 12.5% of the global population affected (1). A recent cohort study further highlighted that patients with severe periodontitis face a 1.3-fold increased risk of all-cause mortality compared to those with no or mild periodontitis (2). Preventing severe periodontitis throughout the life course is crucial to reducing the risk of secondary diseases and associated mortality.

One potential intervention to mitigate the risk of severe periodontitis is quitting the use of combustible cigarettes (CCs). Systematic reviews have shown that individuals with periodontal disease who quit smoking experience significantly less (approximately one-third) clinical attachment loss and radiographic bone loss compared to those who continue smoking (3, 4).

However, the relationship between switching from daily CC smoking to alternative smoking statuses such as heated tobacco products (HTPs) and severe periodontitis remains unclear. HTPs have gained popularity recently among a relatively younger generation, particularly in Japan (5), accounting for approximately 70% of the global HTPs market (6). Unlike CCs, HTPs produce aerosols containing chemicals such as nicotine and glycerol by heating rather than burning tobacco leaves (7, 8). The global prevalence of HTP use is expected to increase in a short time (9).

The use of tobacco products, including CCs and HTPs, has become increasingly diverse. A notable trend is the switching from daily CC smoking to dual use of CCs and HTPs; while only approximately 10% of CC smokers are dual users, dual use constitutes the majority of HTP users, accounting for approximately two-thirds (10). This switching to dual use is often driven by the perception that HTPs are less harmful than CCs (11, 12). However, most safety data on HTPs are from tobacco industry sources (13), and independent evidence on their impact on general and oral health, including periodontal status, remains underexplored. Despite the growing prevalence of HTP use, only one epidemiological study has evaluated the association between dual use of CCs and HTPs and the presence of periodontal conditions, which was cross-sectional in design, suggesting a potential link (14). Furthermore, existing research on the harmful effects of HTPs on periodontal tissues includes just one cross-sectional study and three *in vitro* studies, which reported outcomes such as increased probing depth and damage to gingival epithelial and ligament cells (15–18). Only a cross-sectional study has examined the association between HTP use or dual use and the prevalence of severe periodontitis (14), creating a knowledge gap. Addressing this knowledge gap is critical to prevent severe periodontal conditions.

In the present study, we hypothesized that switching from daily CC smoking to dual use of CCs and HTPs does not reduce the risk of severe periodontitis. This study aimed to assess the association between changes in smoking status [continuous CC smoking, dual use (HTP- or CC-dominant), and quitting or reducing smoking] and worsening periodontal health among Japanese adults.

## 2 Materials and methods

### 2.1 Setting and participants

This longitudinal study used panel data obtained from the Japan COVID-19 and Society Internet Survey (JACSIS; https://jacsis-study.jp/index.html). JACSIS was designed to explore information on lifestyle, health, social, and economic factors, including the impact of COVID-19, through internet-based questionnaires. The pre-baseline survey, conducted in 2021, was distributed to 35,719 panelists registered with a Japanese internet research company between September 27 and October 29, 2021. The 2022 survey followed from September 12 to October 19, and the 2023 survey was conducted between September 25 and November 17, 2023. This study included participants from the 2021 survey who were daily smokers of only CCs and had periodontitis. Individuals under 20 years or over 75 years, as well as those who selected “other” for their educational information, and those whose answers were considered unreliable responses (i.e., the participants answered they had all current chronic disease or drug use listed in the options), were excluded from the analysis.

### 2.2 Outcome variable

The transition from periodontitis in the 2021 survey to severe periodontitis in the 2023 survey was used as the outcome variable in this study. First, periodontitis in the 2021 survey was evaluated using four questions: “Have your gums bled recently?”, “Do you think your gums have receded, making your teeth appear longer than before?”, “Have you ever been told at a dental clinic that you needed treatment for periodontitis or gums issues?”, and a question about smoking status. For the smoking status question, participants were asked whether they currently or previously smoked CCs. Participants were classified as having periodontitis if they answered “Yes” to three or four of these questions. This definition of screening in periodontitis has been reported in a previous study (19). In the current study, the target participants were daily CC smokers in the 2021 survey and were automatically categorized as “Yes” for the smoking status question. Therefore, periodontitis in the 2021 survey was defined for participants who answered “Yes” to at least two of the other three questions regarding periodontal status. Second, severe periodontitis in the 2023 survey was determined using the following four questions: “Do you think you might have periodontitis?”, “Have you ever had a loose tooth?”, “Have you ever been told by a dentist or dental hygienist that you have bone loss around your teeth?” and “Have you experienced gum bleeding in the last 3 months?” Participants were classified as having severe periodontitis if they answered “Yes” to two or more of these questions. This definition of severe periodontitis was validated in a prior study (20).

### 2.3 Exposure variable

The change in smoking status from the 2021 survey to the 2022 survey was used as the exposure variable. Both surveys included the same question regarding the frequency and type of tobacco products participants smoked or used: “Have you currently smoked or used each tobacco product?” Participants selected one of the following responses: “I have never smoked or used.”, “I smoked or used more than once but have not used routinely.”, “I had smoked or used routinely before but quit recently.”, “I smoke or use sometimes.”, or “I smoke or use almost every day.” The tobacco products listed included CCs (hand-rolled tobacco and manufactured cigarettes) and HTPs, which are the predominant tobacco products used in Japan (21). Other tobacco or nicotine products were not included due to their very low prevalence. Additionally, participants were asked how many days they smoked CCs or used HTPs in the past 30 days in both the 2021 and 2022 surveys. First, participants who smoked CCs exclusively for more than 30 days per month in the 2021 survey were defined as “daily CC smokers.” Next, daily CC smokers in the 2021 survey were categorized into four groups based on their smoking status in the 2022 survey: continuous CC smokers (those who continued to smoke only CCs for 30 or more days per month), switching to dual users (HTP-dominant, those who used both CCs and HTPs for 30 or more days per month, with HTPs being used more days per month than CCs), switching to dual users (CC-dominant, those who use both CCs and HTPs for 30 or more days per month, with CCs being used more days per month than HTPs), and quitter/reduced smokers (those who completely quit smoking CCs or reduced their smoking frequency to fewer than 30 days per month).

### 2.4 Covariates

**Figure 1:**
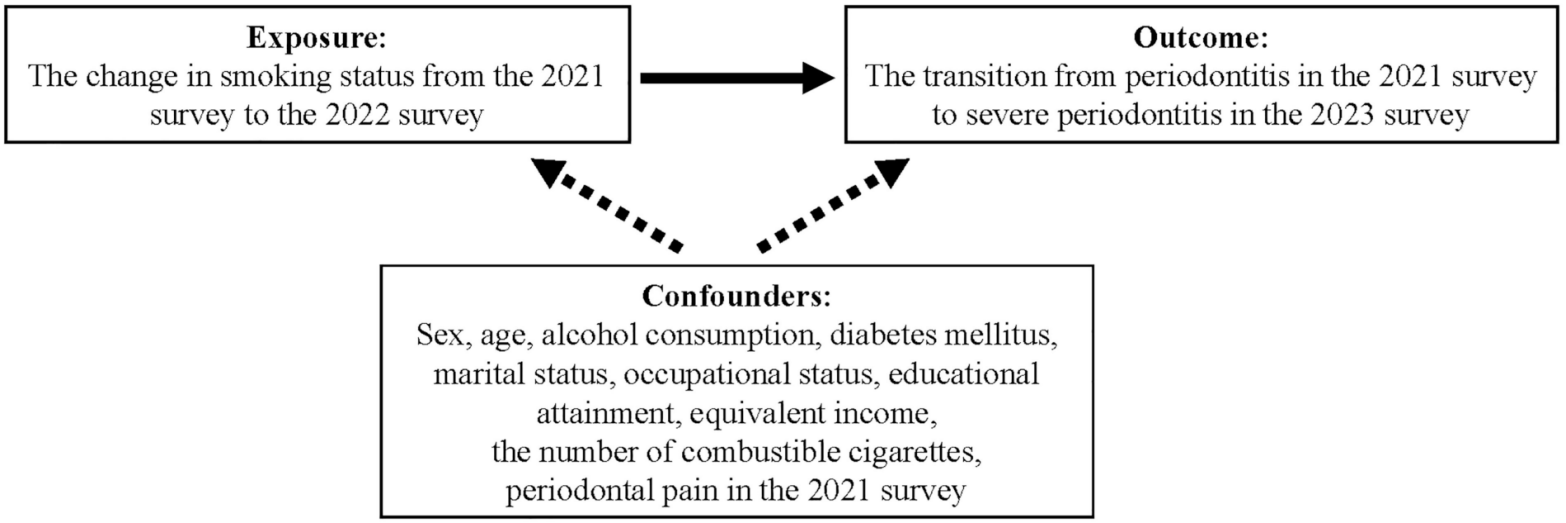
Directed acyclic graph in this study.

Based on previous studies and clinical knowledge (14), the following variables were included as covariates: sex (male/female), age, educational attainment (junior high school/high school, vocational/technical college/junior college, or university/graduate school), occupational status (employer or unemployed), marital status (married, never married, or widowed/divorced), equivalent income (<14,000 USD, 14,000–28,000 USD, ≥28,000 USD, or declined to answer), alcohol consumption (never, former, or current drinker), diabetes mellitus status (no or yes), periodontal pain (no or yes), and smoking frequency (number of days per month the participants smoked CCs in the 2021 survey). For smoking frequency, we included the number of days the participants smoked CCs per month measured in the pre-baseline survey (2021) as a confounding variable. Smoking intensity measured at the baseline survey (2022) was not included because it was assessed concurrently with exposure and therefore did not precede it; thus, baseline smoking intensity could not be considered a prior confounding factor (Figure 1). Periodontal pain has been previously reported as a proxy indicator of periodontal disease severity (22). Therefore, periodontal pain from the 2021 survey was included to better account for disease severity at pre-baseline.

### 2.5 Statistical analysis

We employed the modified Poisson regression model with robust sandwich standard error to estimate risk ratios (RRs) and 95% confidence intervals (CIs) (23), evaluating the association between changes in smoking status from 2021 to 2022 and the transition from periodontitis in 2021 to severe periodontitis in 2023. We constructed both crude and adjusted models, with all covariates included in the adjusted analyses. Statistical significance was set at alpha = 0.05.

To address potential selection bias, we conducted a sensitivity analysis using inverse probability weighting (IPW) (24). Propensity scores were estimated using the covariates included in the primary analyses. Weighted modified Poisson regression models were then fitted to evaluate the robustness of the findings.

A directed acyclic graph (DAG) in Figure 1 was used to visualize the assumed causal structure among the exposure, confounders, and outcome. All statistical analyses were performed using Stata/MP version 18.0 (Stata Corp., College Station, TX, USA).

## 3 Results

**Figure 2:**
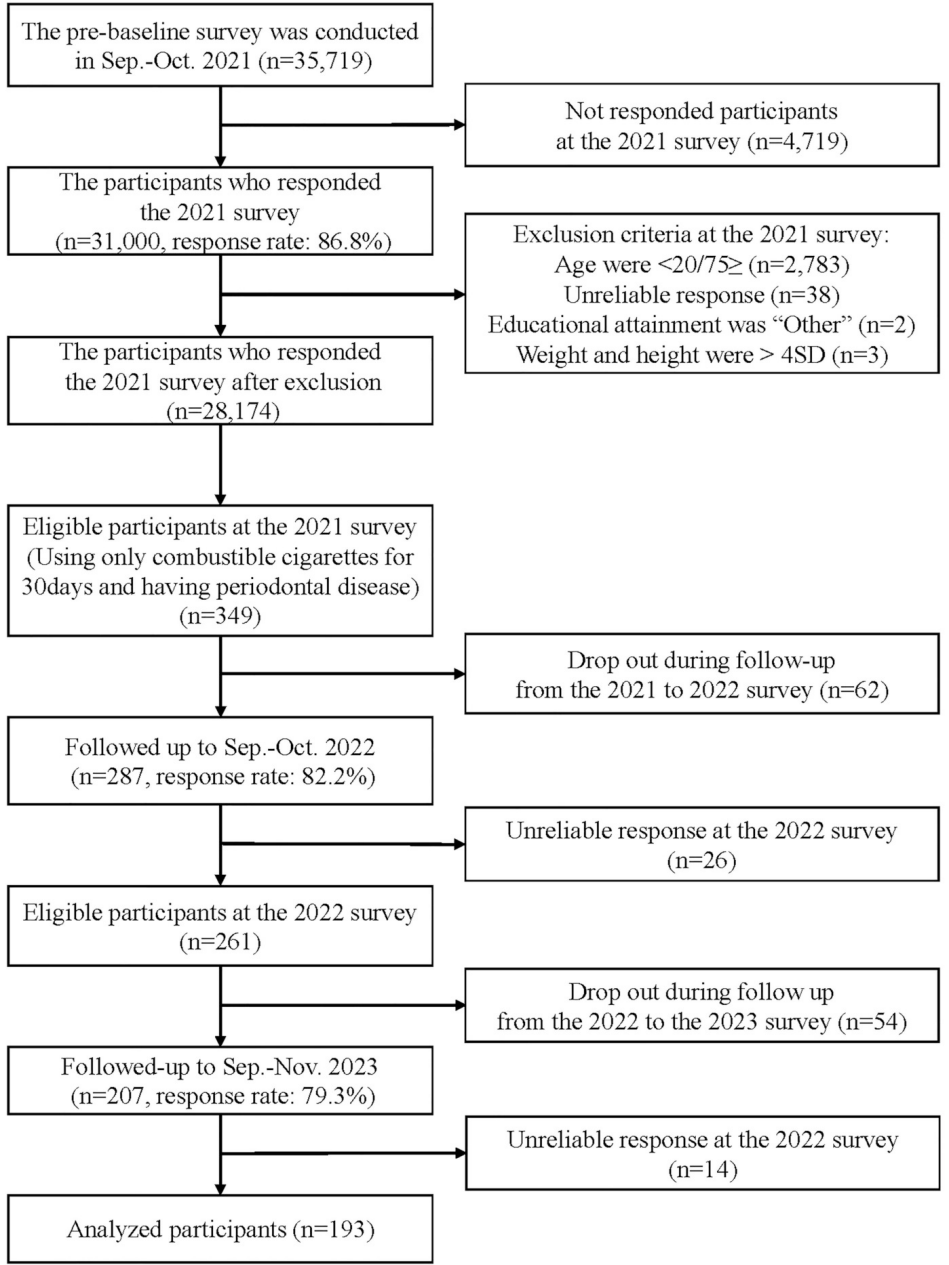
Participants flowchart for the analytic sample (*n* = 193).

Figure 2 shows a flow chart illustrating the inclusion process for the study participants. A total of 193 participants were included in this analysis. Table 1 presents the characteristics of the participants. The participants had a mean age of 57.6 years (SD = 10.9), and 67.9% were male. After a 2-year follow-up period, 61.1% (*n* = 118) of the participants had severe periodontitis. Between the 2021 and the 2022 survey, the proportions of participants who continued smoking only CCs, switched to dual use (HTP-dominant), switched to dual use (CC-dominant), or quit/reduced smoking CCs were 67.4% (*n* = 130), 4.1% (*n* = 8), 3.6% (*n* = 7), and 24.9% (*n* = 48), respectively.

**Table 1:** Descriptive characteristics of the participants.

Variables	All participants		Severe periodontitis^a^			
			No		Yes	
	*n*	%	*n*	%	*n*	%
Total	193	100	75	38.9	118	61.1
Baseline smoking status^b^						
Continuous CC smoker	130	67.4	52	40	78	60
Dual user (HTP-dominant)	8	4.1	3	37.5	5	62.5
Dual user (CC-dominant)	7	3.6	1	14.3	6	85.7
Quitter/reduced smoker	48	24.9	19	39.6	29	60.4
Age^c^	57.6	(10.9)	55.3	(12.0)	59	(9.9)
The number of CC smoking days^d^	30.3	(2.8)	30	(0)	30.5	(3.6)
Sex						
Male	131	67.9	49	37.4	82	62.6
Female	62	32.1	26	41.9	36	58.1
Alcohol consumption						
Non-current	75	37.3	23	59.6	49	65.3
Current	121	62.7	52	43	69	57
Diabetes mellitus						
No	172	89.1	70	40.7	102	59.3
Yes	21	10.9	5	23.8	16	66.2
Marital status						
Married	116	60.1	43	37.1	73	62.9
Never married	40	20.7	17	42.5	23	57.5
Widowed/divorced	37	19.2	15	40.5	22	59.5
Occupational status						
Unemployed	65	33.7	18	27.7	47	72.3
Employer	128	66.3	57	44.5	71	55.5
Educational attainment						
Junior high school/high school	74	38.3	28	37.8	46	62.2
Vocational/technical college/junior college	42	21.8	21	50	21	50
College/university/graduate school	77	39.9	26	33.8	51	66.2
Equivalent income (USD)						
<14,000	33	17.1	14	42.4	19	57.6
14,000–28,000	73	37.8	26	35.6	47	64.4
≥28,000	50	25.9	24	48	26	52
Decline to answer	37	19.2	11	29.7	26	60.3
Periodontal pain						
No	125	64.8	56	44.8	69	55.2
Yes	68	35.2	19	27.9	49	72.1

CC, combustible cigarettes; HTP, heated tobacco products; USD, United States Dollars.

a The transition to severe periodontitis is based on the change from the 2021 survey to the 2023 survey.

b Baseline smoking status is based on the 2022 survey.

c Age and the number of days the participants smoked CC are shown in mean and standard deviation.

d The number of CC smoking days is based on the 2021 survey.

Table 2 shows the findings of the modified Poisson regression analysis. After adjusting for all covariates, the risk of severe periodontitis was 1.19 times (95% CI: 0.67–2.11) higher among dual users of the HTP-dominant type and 1.41 times (95% CI: 1.04–1.89) higher among dual users of CC-dominant type compared to continuous CC smokers. However, no significant difference was observed between quitters/reduced smokers and continuous CC smokers (RR = 0.99; 95% CI: 0.76–1.28). Similar findings were observed in the sensitivity analysis, which adjusted for dental treatment during the follow-up period (Table 3).

**Table 2:** The association between the change of smoking status and the risk of severe periodontitis.

Exposure variable	Risk of severe periodontitis	
	Crude model	Adjusted model
	RR (95% CI)	RR (95% CI)^a^
Smoking status		
Continuous CC smoker	1.00 (reference)	1.00 (reference)
Dual user (HTP-dominant)	1.04 (0.60–1.82)	1.19 (0.67–2.11)
Dual user (CC-dominant)	1.43 (1.02–2.00)*	1.41 (1.04–1.91)*
Quitter/reduced smoker	1.01 (0.77–1.32)	0.99 (0.76–1.28)

CC, combustible cigarettes; CI, confidence interval; HTP, heated tobacco products: RR, risk ratio.

a Adjusted for age, sex, drinking alcohol, diabetes, marital status, education level, equivalent income, periodontal pain, and the number of days the participants smoked CC in 2021.

* *p* < 0.05.

**Table 3:** The association between the change of smoking status and the risk of severe periodontitis with inverse probability weighting.

Exposure variable	Risk of severe periodontitis	
	Crude model	Adjusted model
	RR (95% CI)	RR (95% CI)^a^
Smoking status in baseline		
CC daily smoker	1.00 (reference)	1.00 (reference)
Dual user (HTP-dominant)	1.04 (0.60–1.82)	1.11 (0.60–2.05)
Dual user (CC-dominant)	1.43 (1.02–2.00)*	1.56 (1.29–1.88)**
Quitter/reducer	1.01 (0.77–1.32)	0.97 (0.73–1.30)

CC, combustible cigarettes; CI, confidence interval; HTP, heated tobacco products; RR, risk ratio.

a Adjusted for age, sex, drinking alcohol, diabetes, marital status, education level, equivalent income, periodontal pain, and the number of days the participants smoked CC in 2021.

* *p* < 0.05.

** *p* < 0.001.

## 4 Discussion

### 4.1 Summary of main findings

This prospective cohort study demonstrated that switching from daily CC smoking to dual use of CCs and HTPs was associated with an increased risk of severe periodontitis. However, no significant association was observed between transitioning from daily CC smoking to quitting or reducing smoking and the risk of severe periodontitis. Notably, the findings indicated that switching from daily CC smoking to dual use of CCs and HTPs may increase the risk of severe periodontitis rather than reduce it.

### 4.2 Comparison with previous studies

Our findings align with those of a previous cross-sectional study, which reported that dual users of CCs and HTPs had a higher prevalence of periodontal disease compared to CC-only smokers, using non-smokers as the reference group (14). The increased risk of periodontal disease in dual users of CCs and HTPs compared to CC-only users may be attributed to multiple factors. For individuals who switched from daily CC smoking to dual use of CCs and HTPs, their periodontal health is likely influenced by the amount of CCs they smoked in the baseline survey and the additional toxic effects of HTP use (15–18). Consequently, the dual use of CCs and HTPs may exacerbate the risk of severe periodontitis, making it worse than for CC-only smokers. Moreover, the difference in the risk of severe periodontitis between dual users of HTP-dominant and CC-dominant types in our study could be explained by differences in CC exposure. Dual users of CC-dominant type smoked CCs for an average of 30.6 days, approximately 10 days longer than the average 20.0 days for dual users of HTP-dominant type in the baseline (2022) survey. This greater exposure to CC smoke is thought to be linked to the higher risk of severe periodontitis among dual users of CC-dominant type.

In contrast, our study found no significant difference in the risk of severe periodontitis between quitters/reduced smokers and continuous CC smokers. These findings partially differ from previous studies, which reported a lower prevalence of periodontal disease among individuals who had quit smoking for longer periods (25–27). A possible explanation for this discrepancy is the shorter duration of smoking cessation or reduction in our study, which was limited to 1 year, compared to the 10 years or more considered in earlier studies. Future research involving longer durations of smoking cessation or reduction may reveal a lower risk of severe periodontitis among quitters/reduced smokers compared to continuous CC smokers.

### 4.3 Clinical implications from the study

Our findings suggest that switching to dual use of CCs and HTPs can have harmful effects on periodontal tissues, similar to those caused by CC smoking. Previous cohort studies have demonstrated that switching to dual use may increase the likelihood of relapse into regular smoking (28) and is not effective in supporting smoking cessation (29). From a clinical dentistry perspective, this highlights the importance of complete smoking cessation, rather than merely switching, to preserve and improve periodontal health. In addition, earlier studies have shown that periodontal treatment alone may not be effective in improving periodontal health for smokers (30, 31). Therefore, a combined approach that includes both smoking cessation therapy and dental treatment by dental professionals is essential for the effective management of periodontal health.

### 4.4 Limitations and strengths of the study

Our study has some limitations. First, the use of self-reported questionnaires may have introduced information bias. The measurements for smoking status (exposure) and periodontitis (outcomes) were based on subjective responses, which might not accurately reflect the actual conditions and could lead to exposure or outcome misclassification. To mitigate this concern, we adopted previously validated definitions of periodontitis and severe periodontitis and additionally adjusted for periodontal pain to account for baseline disease severity. Future research incorporating clinical assessment for periodontal status and detailed smoking status data alongside self-reported questionnaires could strengthen these findings. Second, regarding confounding, although we accounted for various potential confounders, unmeasured factors, such as the number of years the participants smoked CCs before the survey and the duration of smoking, may have influenced the results. However, we have attempted to address this issue by adjusting for the number of days participants smoked CCs before the baseline. Third, we used the number of days participants smoked CCs and used HTPs as a measure of CCs and HTPs consumption. However, future studies should incorporate more detailed data, such as the exact number of CCs and HTPs sticks consumed, to better clarify the association. Fourth, regarding generalizability, this study was based on an internet survey, and the participants could not be representative of national data. However, the national survey reported that 69.7% of current smokers in men and 63.2% of current smokers in women smoked only CCs in Japan (21), which is similar to our study participants, continuous CC smokers (67.4%). Finally, the strict inclusion criteria resulted in a relatively small analytic sample (*n* = 193), which may generate selection bias. We addressed this point by presenting a DAG and conducting sensitivity analyses using IPW, both of which supported the robustness of our main findings.

Our study has some notable strengths. First, we developed a comprehensive smoking trajectory framework, focusing on daily CC smokers, defined as individuals who smoked CC every day prior to the baseline. These participants were categorized into distinct smoking patterns, including switching dual users of HTP/CC-dominant type, based on the reported frequency of CC and HTP use in the baseline survey. Second, to the best of our knowledge, this is the first longitudinal study to assess the association between switching from daily CC smoking to dual use of CCs and HTPs and the risk of severe periodontitis. This longitudinal design provides valuable new evidence on the severity of periodontitis in relation to smoking status while addressing the potential for reverse causation.

## 5 Conclusion

This study highlights the potential role of switching from daily CC smoking to particularly CC-dominant dual use of CCs and HTPs in increasing the risk of severe periodontitis. The findings emphasize that combining CCs with HTPs does not constitute a harm-reduction approach for periodontal health. Dual use, regardless of combustible cigarette frequency, should therefore not be recommended in dental or public health practice for preventing severe periodontitis.

## Data Availability

The data analyzed in this study is subject to the following licenses/restrictions: The data used in this study are not available in a public repository because they contain personally identifiable or potentially sensitive patient information. Based on the regulations for ethical guidelines in Japan, the Research Ethics Committee of the Osaka International Cancer Institute has imposed restrictions on the dissemination of the data collected in this study. Requests to access these datasets should be directed to all data enquiries should be addressed to the person responsible for data management, Dr. Takahiro Tabuchi at the following e-mail address: tabuchitak@gmail.com.
